# Raman microspectroscopy and machine learning for use in identifying radiation-induced lung toxicity

**DOI:** 10.1371/journal.pone.0279739

**Published:** 2022-12-30

**Authors:** Ramie N. Ali-Adeeb, Phil Shreeves, Xinchen Deng, Kirsty Milligan, Alex G. Brolo, Jullian J. Lum, Christina Haston, Jeffrey L. Andrews, Andrew Jirasek

**Affiliations:** 1 Department of Physics, The University of British Columbia - Okanagan campus, Kelowna, BC, Canada; 2 Department of Statistics, The University of British Columbia - Okanagan campus, Kelowna, BC, Canada; 3 Department of Chemistry, University of Victoria, Victoria, BC, Canada; 4 Department of Biochemistry and Microbiology, University of Victoria, Victoria, BC, Canada; 5 Trev and Joyce Deeley Research Centre, BC Cancer, Victoria, BC, Canada; Indian Institute of Space Science and Technology, INDIA

## Abstract

**Objective:**

In this work, we explore and develop a method that uses Raman spectroscopy to measure and differentiate radiation induced toxicity in murine lungs with the goal of setting the foundation for a predictive disease model.

**Methods:**

Analysis of Raman tissue data is achieved through a combination of techniques. We first distinguish between tissue measurements and air pockets in the lung by using group and basis restricted non-negative matrix factorization. We then analyze the tissue spectra using sparse multinomial logistic regression to discriminate between fibrotic gradings. Model validation is achieved by splitting the data into a training set containing 70% of the data and a test set with the remaining 30%; classification accuracy is used as the performance metric. We also explore several other potential classification tasks wherein the response considered is the grade of pneumonitis and fibrosis sickness.

**Results:**

A classification accuracy of 91.6% is achieved on the test set of fibrotic gradings, illustrating the ability of Raman measurements to detect differing levels of fibrotic disease among the murine lungs. It is also shown via further modeling that coarser consideration of fibrotic grading via binning (ie. ‘Low’, ‘Medium’, ‘High’) does not degrade performance. Finally, we consider preliminary models for pneumonitis discrimination using the same methodologies.

## Introduction

Cancers treated with radiation in the thoracic region are limited by the occurrence of radiation induced lung toxicity, which can occur in up to 15–30% [1–3] of patients after treatment. Generally, the pathologies can be characterized by excessive inflammation (pneumonitis) or the deposition of extracellular matrix/collagen in the interstitium of the lung (fibrosis). Furthermore, lung toxicity can occur months to years after exposure resulting in impaired lung function and, in some cases, respiratory failure [3].

To study the toxicity response observed clinically, the radiation-induced pneumonitis and fibrosis response has been modelled in mice which have strain and genotype dependent presentations of these traits [4–9]. It has been documented [7, 8, 10, 11] that certain inbred mice will present respiratory distress at various times after thoracic irradiation with some being more prone to penumonitis while others are more likely to develop fibrosis. Specifically relevant to this work, lethal pneumonitis develops 10–14 weeks following thoracic irradiation in C3H/HeJ mice and pneumonitis with fibrosis develops at 22 weeks or later in C57BL/6J mice.

Raman spectroscopy (RS) is an optical technique based on the inelastic scattering of light upon interaction with matter. This results in a unique “fingerprint” which is dependent upon the chemical composition of the material under investigation. The main advantages of RS for use in biological applications centre around the non-invasive, label-free nature of the technique. Although a fairly weak phenomenon, RS has been used extensively in many studies to distinguish between cancerous and non-cancerous tissue [12] in breast cancers [13–16], skin cancers [17–20], lung cancers [21], brain cancers [22, 23], and many other types of cancerous tissue [24–29]. More recently, RS has proved to be a useful tool in surgical guidance technology, where Zuniga *et al.* [30] demonstrated the use of RS as an efficient method in measuring surgical margins in tumour excisions of breast cancer patients. This can be enormously beneficial in ensuring all cancerous tissue is removed during surgery [31, 32].

Radiation effects have also been studied using RS. It has been previously demonstrated that radiation induced metabolic response pathways can be identified using RS [33–37]. Identification of metabolic pathways that are potentially involved in radiation resistance is enormously beneficial in terms of delivering a more successful treatment plan as radio sensitising drugs can be identified and used in combination therapies [34, 38, 39]. Raman spectroscopy has also been used to create mapped profiles in bronchial and lung tissues [40–42] as well as evaluate tissue pathologies such as liver fibrosis, human skin pilomatrixoma, and oncogenesis in the cervix [43–45]. Taken together, the capabilities of Raman spectroscopy make it an ideal tool for use in biomedical analysis.

Due to the complexity of Raman spectral data, typical methods for analysis involve the application of dimensionality reduction techniques such as principal component analysis (PCA) [33–35, 46, 47], allowing for the decomposition and interpretation of biochemical changes to be measured by RS. As an alternative approach, the successful application of non-negative matrix factorization (NMF) to spectral data sets has been shown to afford advantages over other dimensionality reduction techniques like PCA [36, 48–50]. One of the main advantages of NMF is the linearly additive, factor based representations of a non-negative data matrix which results in increased interpretability, particularly in the context of spectral data as it is representative of spectral superposition [51–53].

In this manuscript, we investigate the utility of RS for use in discrimination between healthy and fibrotic lung tissue. Currently, fibrosis is most commonly diagnosed through pathological examinations and immuno-histochemical (IHC) staining [54, 55]. Although these techniques prove successful in identifying fibrotic tissue, they can be both time-consuming and subjective with respect to different persons carrying out the analysis of tissue sections. RS can provide large multiplexed biological information concerning multiple regions of lung tissue, which is often difficult to achieve using single IHC analyses, resulting in more efficient use of patient samples. Combining RS with both supervised and unsupervised machine-learning techniques, such as NMF, has the potential to create a rapid and robust method of diagnosing and distinguishing fibrotic tissue from healthy lung tissue [43].

The current aim of this work is to utilize RS to identify the formation of radiation-induced murine lung toxicity post radiation therapy. Throughout the following sections, we utilize RS in combination with machine learning techniques to, ultimately, generate models to differentiate between healthy and diseased lung tissue.

## Materials and methods

### Murine model

Mice: C57BL/6J and C3H/HeJ mice were purchased from the Jackson Laboratory (Bar Harbor, ME) and housed in the animal facility of the University of British Columbia—Okanagan. All mice were handled according to protocol A18–0140 approved by the Animal Care Committee at the University of British Columbia, in accordance with regulations set by the Canadian Council on Animal Use and Care.

### Murine irradiation

Eight week old mice were treated with 14 Gy whole thorax irradiation at the BC Cancer Agency—Sindi Ahluwalia Hawkins Centre for the Southern Interior. Mice were anesthetized with intraperitoneal (i.p.) injections of sodium pentobarbitol (30 mg/kg) and xylazine (5 mg/kg). Upon reaching surgical level of anesthesia, mice were placed in a perspex box with a 13 mm thick lid and were irradiated with a flattening filter free, 6 MV Varian Linear Accelerator (Varian, Palto Alto, CA). Field was accurately positioned over thoracic cavity using a kV x-ray.

### Tissue harvesting

After irradiation, mice were monitored weekly until ∼ 10 weeks post treatment for C3H/HeJ and ∼ 20 weeks post-treatment for C57BL/6J mice and subsequently were monitored semi weekly or daily for indications of physical distress as a result of the lung disease development. The experimental endpoint used to define the parameters for humane termination for the mice were ≥20% body weight loss (from greatest observed weight) and/or shallow and rapid breathing, hunching, slow and tip-toe movement, ungroomed fur, and decreased physical responsiveness. The control mice (N = 2 for each strain), were sacrificed with lethal dose of sodium pentobarbitol using standard operating procedures to reduce suffering at the experiment end date(∼ 15 weeks for C3H/HeJ and ∼ 25 weeks for C57BL/6J).

### Lung cryopreservation

Once a mouse qualified for humane endpoint, it was sacrificed with a lethal intraperitoneal injection of sodium pentobarbitol. After breathing ceased, cervical dislocation was performed and the lungs were removed. The left lobe was filled with a 1:1 dilution of optimal cutting temperature solution (TissueTek OCT; Sakura Finetek USA, Inc.) and 1X phosphate buffer solution (PBS; pH 7.4) to expand the lung to maintain anatomical morphology. The expanded lung was then placed in a plastic mold and surrounded by undiluted OCT. The mold was placed on crushed dry ice to freeze.

### Sample processing

Tissue blocks were sectioned using a rotary cryostat (MICROM HM 550) and cuts were made (∼ 50 microns) until the center of the frozen lung was reached. One 20*μ*m section was placed on magnesium fluoride slides (*MgF*_2_) and an adjacent 5*μ*m section was taken for histology. Histology sections were placed on glass slides and stored in a dry ice cooler for transport and storage.

### Pathological grading

Adjacent lung slices were sent to the University of British Columbia Vancouver Histology Lab where they were thawed and chemically fixed with formalin for staining. Following standard histology protocols, one section was stained with haemotoxylin and eosin (H&E) to visualize microscopic anatomy while the other section was stained with Mason’s TriChrome to highlight connective tissue, specifically collagen. To quantify degree of fibrosis phenotype expression, Trichrome stained sections were assessed in accordance with previously established protocols [56]. To summarize, areas determined to be fibrotic, characterized by condensed tissue and collagen build up, were identified, summed, and divided by the total area of the lung.

The H&E stained sections were used to assess the degree of pneumonitis using a semi-quantitative method established in previous work [6, 8, 56]. Sections were inspected and subjectively scored on a scale from 0 to 6 where 0 was no pathology characteristics observed and 6 was extreme pathology development was observed in the form of acute thickening of alveolar walls, high levels of infiltration from immune system cells, and excessive amounts of cellular exudate filling the alveolar spaces throughout the entire lung [8]. Assessment of stained slides was completed on a Motic BA210 microscope at 50x and 100x magnification.

**Fig 1:**
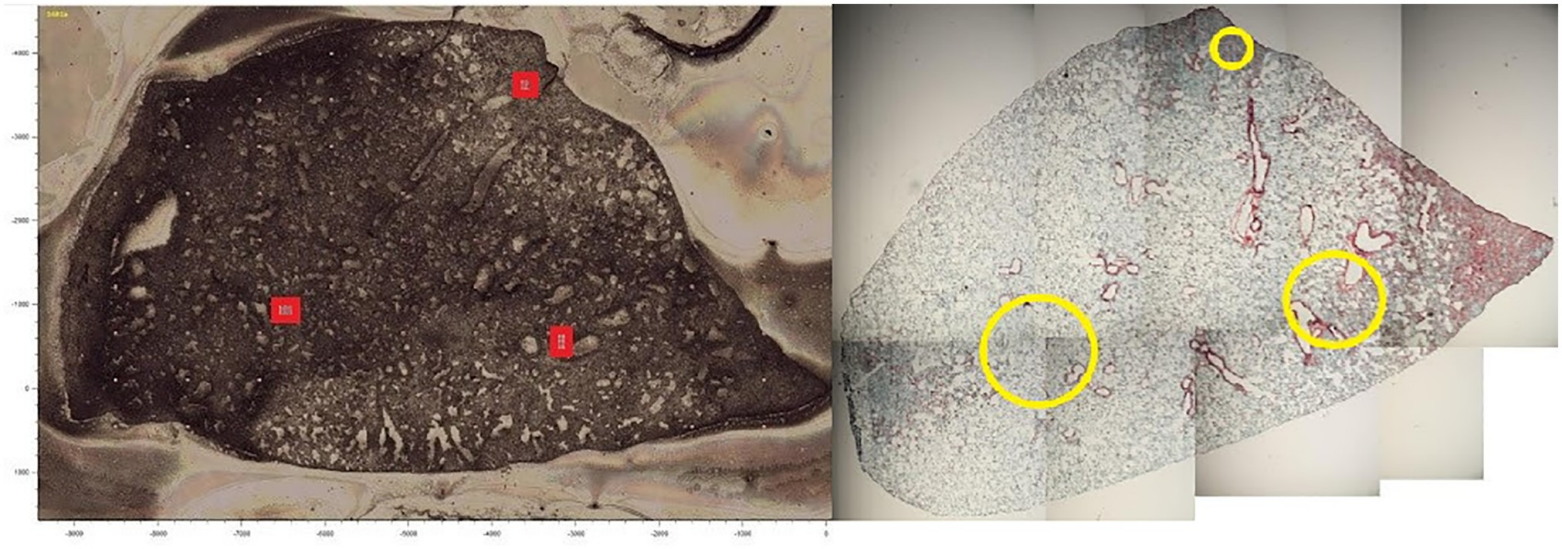
Region matching between Raman bright field images and IHC images. Left: Image of murine lung showing region of Raman acquisition maps (red squares). Right: Mouse lung showing corresponding regions(yellow circles) where pathological grades were assessed.

In order to increase the resolution of the pathology grades from whole lung to specific areas in the lung, Raman map locations (Fig 1A) where matched as accurately as possible by eye to images taken of the histology stains of adjacent lung sections (Fig 1B). In trying to match features in the lung, arbitrary areas were drawn and tissue contained by area was graded by percent of disease present excluding air pockets. Ultimately, the more localized pathology grades were used in the final analysis.

### Raman spectral acquisition

Re-frozen samples were allowed to thaw for 15—20 minutes before being gently rinsed with PBS to remove any excess OCT remaining in and around the sample. After rinsing with PBS, the samples were allowed to air dry for 15—20 minutes before spectral acquisition. Total time for Raman maps across two tissue samples to be acquired ranged from 6—8 hours. RS measurements were performed on an inVia Raman microscope (Renishaw Inc., Illinois, IL USA) with a 100X dry objective (N.A. = 0.9) (Leica Microsystems, Wetzlar, Germany) and a 830 lines/mm or 1200 lines/mm diffraction grating. A 785 nm continuous wave diode laser (300mW max power, ∼ 35mW power at sample, Renishaw Inc) was used for sample excitation with a laser power density of ∼ 0.5 mW/*μ*m^3^ at the sample and a sampling volume of ∼ 100*μ*m^3^.

From each lung section, spectra was acquired from ∼ 3 regions of interest (ROI) with a mapping grip size of 15x15 *μ*m^2^, the location of which were chosen at random across the lung. Generally, each region would consist of 30 − 80 points per map with the goal of ∼ 160 points per lung section. Each point in the grid is a single spectral observation collected for 30 seconds. A 830 lines/mm grating was used for the majority of the data collection (Data set A). A small portion of the data was collected with a 1200 lines/mm grating with the same acquisition parameters. To merge the two data sets, the spectral window of both data sets were reduced to [461—1606 cm^−1^] and Data set B was interpolated using a shape-preserving piece wise cubic interpolation method to match Data set A (MatLab r2018b).

### Data processing

Cosmic rays were removed within the Renishaw instrument software (WiRE, Renishaw Inc). High frequency noise was removed from the spectra using a Savitzky-Golay (SG) filter [57] (window size of n = 3, polynomial order p = 1).

The baseline estimation uses an algorithm based on the Schulze signal removal method [58]. The algorithm first does a rough estimate of the baseline by applying a first order SG filter with a window size of 7% of the total spectra range. The baseline estimation process was repeated for 20 iterations as any further iterations were found to make no significant difference in the estimation.The data was normalized with respect to the area under the curve (AUC) of each spectrum. A Gaussian curve was fitted and centered on the most prominent peak (phenylalanine (Phe); ∼ 1005 cm^−1^) for the first spectrum in a trial set and subsequent data was shifted accordingly to match to address any minor x-axis calibration drift. This was applied across the entire data set for two full iterations. This is the last step in the processing of the data in preparation for statistical analysis.

### Non-negative matrix factorization

Non-negative matrix factorization (NMF) [51] is the process of decomposing a non-negative data matrix of interest into two lower rank non-negative matrices. This approach leads to each observation being represented as an additive linear combination of a common factor set. Here, the lung data of interest is decomposed into matrices H and W (*X* ≈ *WH*), which have been shown to represent the chemical bases that the molecules are composed of [36] and the amount these bases contribute to each spectrum, respectively. As such, each individual spectrum can be written as a linear combination of the chemical bases acquired. A constrained form of NMF, known as group and basis restricted non-negative matrix factorization (GBR-NMF) [59], was used in order to allow for constraining of the factors in the model. The factors specified include both a raw spectrum of the OCT-PBS solution, as well as both averages of 10 quality spectra (i.e. regions uncontaminated by air pockets) and 10 undesirable (i.e. air-pocket or OCT:PBS-contaminated) spectra. The following sections show that the GBR-NMF method is a useful way to remove observations from the data set that may be masked by the OCT-PBS solution.

### Sparse logistic regression

Least absolute shrinkage and selection operator (LASSO) [60] regression models are commonly used in high dimensional analyses as they provide a form of variable selection. This is done by adding a penalty term to the optimization criterion, which tends to shrink the size of the coefficients in the model; even forcing some of them to zero. The severity of the penalization term can be adjusted by the user, allowing the number of coefficients to increase or decrease with respect to said term. This is effectively equivalent to constraining $\sum_{i=1}^{j}|\beta_{i}|$ such that it must be less than or equal to some specified value *s* [61]. Herein, we use the logistic regression implementation of LASSO in order to predict disease in observations using only the Raman spectra acquired throughout, without any other form of data transformation other than the post-processing of the spectral data set described above.

### Model validation and evaluation measures

We first applied the GBR-NMF method to the 4068 × 678 data set using a rank of q = 3. These 3 factors include the average quality spectrum, the average undesirable spectrum, and a raw spectrum of OCT-PBS solution described in Results: Air pocket removal. Observations were then removed from the data set according to a pre-determined spectrum score threshold of ≤1 for the quality spectrum and ≥1 for the media score. The remaining “valid” spectra were then used to predict toxicity grade with respect to the spectra using multinomial LASSO logistic regression. Model performance is evaluated using classification rate, which is expressed as

$$\begin{array}{c} CR=\frac{1}{n}\sum_{i=1}^{n}1\left({\hat{g}}_{i}=g_{i}\right), \end{array}$$ (1)

where **1**() is the indicator function and ${\hat{g}}_{i}$ and *g*_*i*_ are the predicted and true groups of observation *i*. The LASSO penalty term was chosen using 10-fold cross validation with the largest term yielding a multinomial deviance within 1 standard error of the minimum as the objective; the formula and plots for which can be found in Supporting Information. Our main results for fibrotic grading prediction are found through a traditional training-testing setup. Therein, 70% of the observations were randomly retained to train the model and the remaining 30% were used to evaluate the expected classification rate. Additional models, utilizing the full data, were created with respect to binned pathology grading methods and separate mouse strains.

## Results

### Air pocket removal

**Fig 2:**
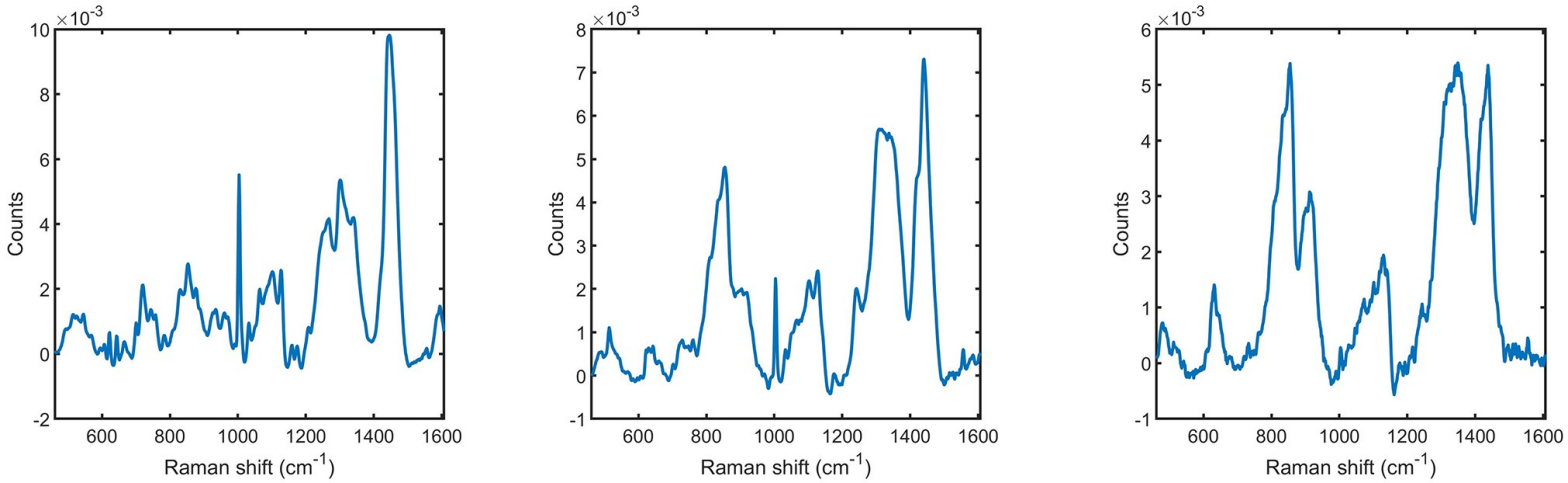
(a) Averaged lung tissue spectrum. (b) Averaged contaminated tissue spectrum. (c) OCT:PBS spectrum (‘media’).

Due to the nature of spectral mapping anatomical sections of the lung, acquiring spectra with little to no tissue present was unavoidable, due to the presence of air pockets. These air pockets contain residual OCT:PBS media which was used to fill the lungs during cryopreservation. To remove these spectral observations from the data set, constrained GBR-NMF was performed using three basis spectra representing RS acquired from lung tissue, lung tissue contaminated with OCT:PBS media (mixed spectrum), and RS of dried OCT:PBS buffer solution, as shown in Fig 2.

As the media spectra were distributed throughout the dataset, it was not feasible to remove all of them by hand. Additionally, there were spectra that resembled both the media and tissue spectrum (example displayed in Fig 2(b)). As such, manual removal would introduce bias into the data as a result of qualitative assessment.

To filter out the air pocket and low quality spectral data, three basis spectra were generated (displayed in Fig 2) and used in GBR-NMF to analyze each spectral acquisition and output a score with respect to each basis. The data was filtered using a threshold of a lung tissue basis spectrum score ≥1 and a media basis spectrum score of ≤1, which are the mean scores of each basis. Spectra falling inside this threshold were kept for further analysis, resulting in 59.4% data retention.

### Prediction of fibrotic grade using Raman spectroscopy and LASSO

**Fig 3:**
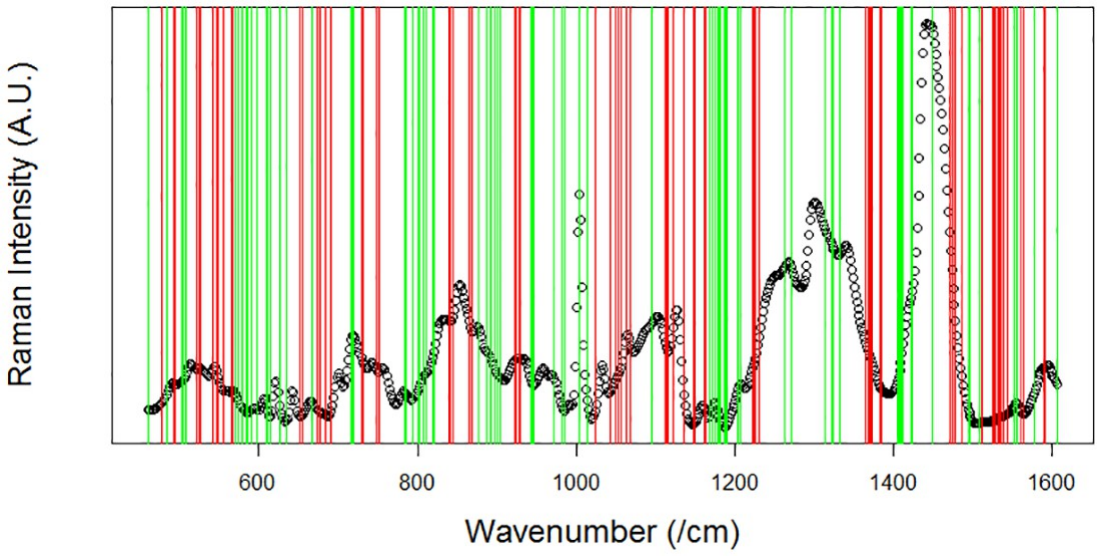
Example of wavenumbers with strong effects in predicting fibrotic grade. These wavenumbers are shown using both red and green lines, with red/green displaying that an increase in intensity results in a decrease/increase in disease likelihood, respectively. White areas are wavenumbers whose coefficients have been reduced to zero.

LASSO logistic regression was applied to the RS data. Based on the LASSO logistic regression model, certain wavenumbers displaying strong effects are retained for disease prediction, while other wavenumbers (coefficients) are reduced to zero in order to simplify the model as a whole; this is illustrated in Fig 3.

Within the LASSO model, disease is not given a binary classification but, rather, each region of observations is taken as a specific grade (probability) between zero and one, representing the proportion of the region considered as diseased. This proportion causes conventional logistic and LASSO logistic regression models to be invalid, as they use response variables of zero and one. While it is possible to create a threshold in the grades, thereby narrowing the classification of observations into either diseased or not, doing so is arguably inappropriate. It cannot be assumed that the individual mouse itself is fully diseased or not diseased at all. For example, suppose a region has a grading of 0.6. This would then mean that roughly 40% of the observations sampled from this region would not have the disease. Therefore, to classify all observations in this region as infected would be misleading. Instead, we suggest that a classification process that has classes other than zero and one should be considered. Herein, multinomial logistic regression is used to classify observations in a multi-class scenario that is very similar to standard logistic regression. We provide the results of applying multinomial LASSO logistic regression for predicting fibrotic gradings to all our tissue spectra in Table 1.

**Table 1:** Multinomial logistic regression on full data set without separating into training and testing sets. Data was classified with respect to pathology gradings of the region the spectra were acquired from.

	Predicted							
	0	0.05	0.1	0.3	0.5	0.6	0.9	1
0	1905	1	6	0	0	0	0	0
0.05	13	30	3	0	0	0	0	0
0.1	17	1	84	7	0	0	0	0
0.3	4	1	1	132	0	0	0	0
0.5	5	0	0	6	13	0	0	0
0.6	2	0	1	1	0	59	0	0
0.9	2	0	0	0	0	0	59	0
1	0	0	0	0	0	0	0	59

To further ensure the validity of the models, the data set was divided into training and testing sets. The randomly selected training set held 70% of the observations and the testing set held the other 30%. Classification results of the model can be found in Table 2.

**Table 2:** Multinomial logistic regression prediction on the testing data set (70% training, 30% testing). Data was classified with respect to pathology gradings of the region the spectra were acquired from.

	Predicted							
	0	0.05	0.1	0.3	0.5	0.6	0.9	1
0	565	2	7	3	0	0	0	0
0.05	8	4	2	0	0	0	1	0
0.1	6	2	20	5	0	1	0	0
0.3	2	1	0	36	1	0	1	1
0.5	3	0	1	1	2	0	0	0
0.6	5	0	2	0	0	10	0	3
0.9	2	0	0	0	0	0	12	1
1	0	0	0	0	0	0	1	13

The multinomial logistic LASSO model performs quite well when distinguishing fibrotic grades, yielding a testing set classification rate of approximately 91.6%. The training classification rate of the model that uses all available data (results shown in Table 1) is, as expected, higher than the testing set results at approximately 96.9%. This is to be expected as the full training model is not making predictions on new data, meaning the model has already seen the spectra it is classifying. The training/testing model, on the other hand, is predicting the class of observations that are not included in the model creation and provides a more believable estimate of the long-run classification rate.

It should also be noted that the pathology grades, either whole lung or regional, are vulnerable to human error. To combat this problem, the regional pathology grades were partitioned into different risk levels, creating a more generalizable approach. The grades at hand (0,0.05,0.1,0.3,0.5,0.6,0.9,1) can be separated using the two different binning methods outlined below:

1. Low (0–0.1), Medium (0.3–0.6), High (0.9–1).
2. Zero (0), Low (0.1–0.3), Medium (0.5–0.6), High (0.9–1).

As expected, both binning methods still yielded excellent results. This is merely a simplification of the models shown in Tables 1 and 2, reducing the number of groups from eight to three and four. Since binned groups also follow a certain order, observations being binned together do have stronger similarities to each other than those in the other groups. Prediction tables of said grouping methods are shown in Tables 3 and 4, which held classification rates of 97.8% and 96.9% respectively. These values are similar to those without the binning process, demonstrating that binning the grades does not degrade the value of the results.

**Table 3:** Multinomial logistic regression binned with respect to fibrotic pathology grades: Low (0–0.1), medium (0.3–0.6), high (0.9–1).

	Predicted		
	Low	Medium	High
Low: 0—0.1	2050	18	1
Medium: 0.3—0.6	21	198	7
High: 0.9—1	3	2	115

**Table 4:** Multinomial logistic regression binned with respect to fibrotic pathology grades: Zero (0), low (0.1–0.3), medium (0.5–0.6), high (0.9–1).

	Predicted			
	Zero	Low	Medium	High
Zero: 0	1898	15	0	0
Low: 0.1—0.3	31	264	1	0
Medium: 0.5—0.6	6	18	62	1
High: 0.9—1	2	0	2	116

### Mouse strain specific analyses using Raman spectroscopy and LASSO

Models were also created with respect to the two individual strains to test how the reduction of variability across strains in the data set may aid the modelling of fibrosis, and even allow for the modelling of pneumonitis. As shown in Tables 5 and 6, classification accuracy improved for the fibrotic gradings (99.1% for C57BL/6 mice only and 99.4% for C3H/HeJ mice only). Although it is only a preliminary model, Table 7 also provides excellent differentiation among the pneumonitis gradings (89.2% for C3H/HeJ mice only). For all three scenarios, given the small sample sizes for some non-zero grades, proper validation of these results via training/testing is left for future work.

**Table 5:** Multinomial logistic regression on spectral data obtained from C57BL/6 mice only. Data was classified based on degree of fibrosis (regional pathology grades).

	Predicted						
	0	0.05	0.1	0.3	0.6	0.9	1
0	1311	1	2	0	0	0	0
0.05	11	35	0	0	0	0	0
0.1	0	0	70	0	0	0	0
0.3	0	0	0	57	0	0	0
0.6	1	0	0	0	62	0	0
0.9	0	0	0	0	0	61	0
1	0	0	0	0	0	0	59

**Table 6:** Multinomial logistic regression on spectral data obtained from C3H/HeJ mice only. Data was classified based on degree of fibrosis (regional pathology grades).

	Predicted			
	0	0.1	0.3	0.5
0	599	0	0	0
0.1	1	37	1	1
0.3	0	0	81	1
0.5	0	0	0	24

**Table 7:** Multinomial logistic regression on spectral data obtained from C3H/HeJ mice only. Data was classified based on degree of pneumonitis (regional pathology grades).

	Predicted							
	0	0.05	0.1	0.15	0.2	0.3	0.5	0.8
0	30	6	6	0	0	0	0	0
0.05	1	223	5	0	0	0	1	0
0.1	4	10	172	6	0	0	0	0
0.15	0	0	6	93	0	0	0	0
0.2	0	0	0	0	35	1	0	0
0.3	0	0	2	2	1	24	11	0
0.5	0	1	1	1	0	1	76	2
0.8	0	0	1	0	1	2	8	12

## Discussion

A variety of LASSO logistic regression models were created in order to ensure robustness and validity of said models. This was done to predict fibrotic disease grade with respect to Raman spectra and was shown to have excellent classification accuracy (96.9% on full training data & 91.6% on the testing set when separated into 70% training and 30% testing).

When further binning the grades, training classification rates (100% of data included in model) of 97.8% and 96.9% were found for binning process 1 and 2 respectively. This result agreed with the initial training classification of 96.9% using the original grading scheme. The first binning process has a slightly higher classification rate than the others, which could likely be attributed to the greater simplicity of the model.

Models were also created for the individual strains of mice. This was to further test how the variability affected the prediction process with respect to disease grades. As shown in Tables 5 and 6, classification rates improved when the strains were separated. Furthermore, modeling C3H/HeJ data alone permitted additional preliminary modelling of pneumonitis grades, which proved challenging when considering both strains together due to added complexities. The modelling of which has been left for future work.

## Conclusion

Using RS in tandem with supervised machine learning techniques, radiation induced lung disease (fibrosis in particular) was effectively measured in a murine model. Looking forward, the methods developed and tested in this work can be applied to explore application in early detection of radiation induced lung toxicity. Ultimately, the goal would be to determine if Raman spectroscopy can predict the development of lung toxicity in murine lung tissue on time frames shorter than current standard of practice.

## Supporting information

S1 Fig Cross validation curve for general data set. (TIFF)

S2 Fig Cross validation curve for train/test data set. (TIFF)

S3 Fig Cross validation curve for binned data—Pathology grades: Low, medium, high. (TIFF)

S4 Fig Cross validation curve for binned data—Pathology grades: Zero, low, medium, high. (TIFF)

S5 Fig Cross validation curve for strain separated data—C57BL/6. (TIFF)

S6 Fig Cross validation curve for strain separated data—C3H/HeJ. (TIFF)

S7 Fig Cross validation curve for strain separated data—C3H/HeJ: Pneumonitis grades. (TIFF)

S1 Appendix Formula for multinomial deviance. (PDF)
