# Supplementary figures and images for: Raman microspectroscopy and machine learning for use in identifying radiation-induced lung toxicity
Source: PLoS One. 2022 Dec 30;17(12):e0279739. doi: 10.1371/journal.pone.0279739 (PMC9803148; doi:10.1371/journal.pone.0279739)

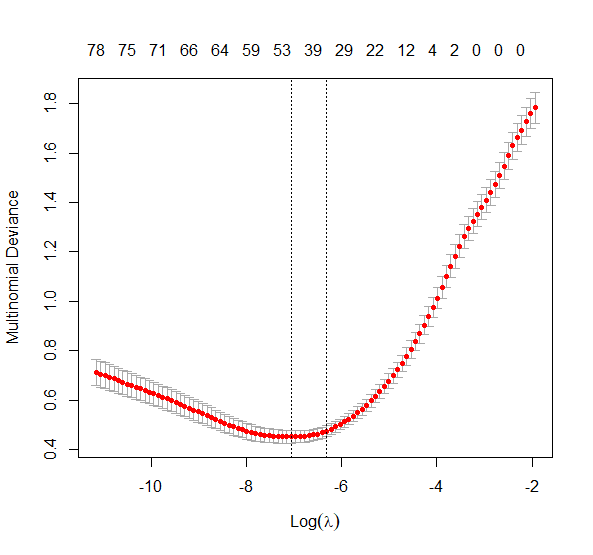

Supplement: S1 Fig — (TIFF) [file pone.0279739.s001.tiff]

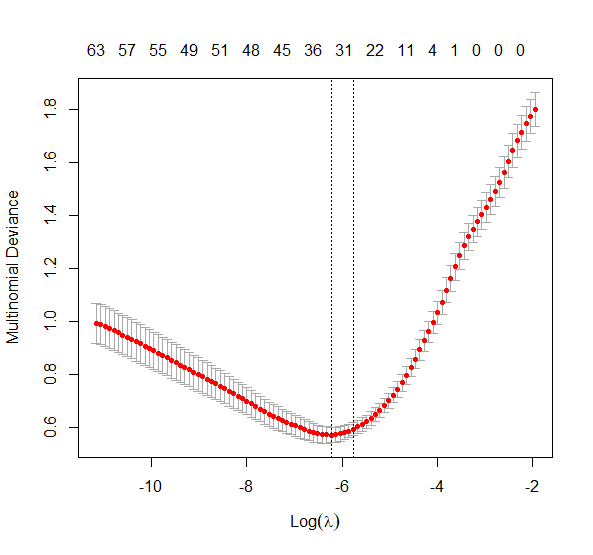

Supplement: S2 Fig — (TIFF) [file pone.0279739.s002.tiff]

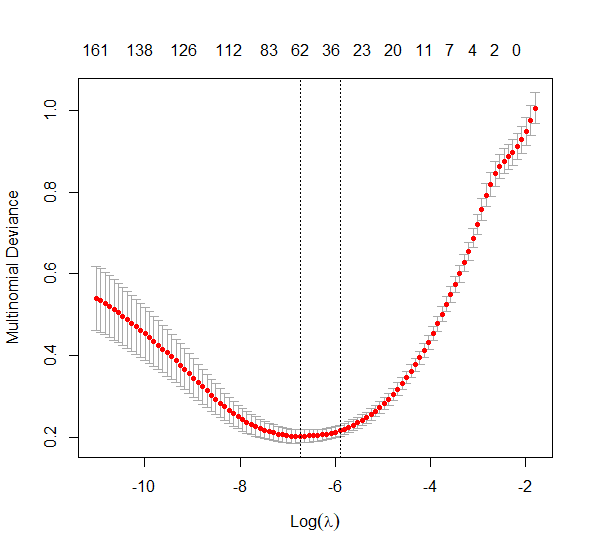

Supplement: S3 Fig — (TIFF) [file pone.0279739.s003.tiff]

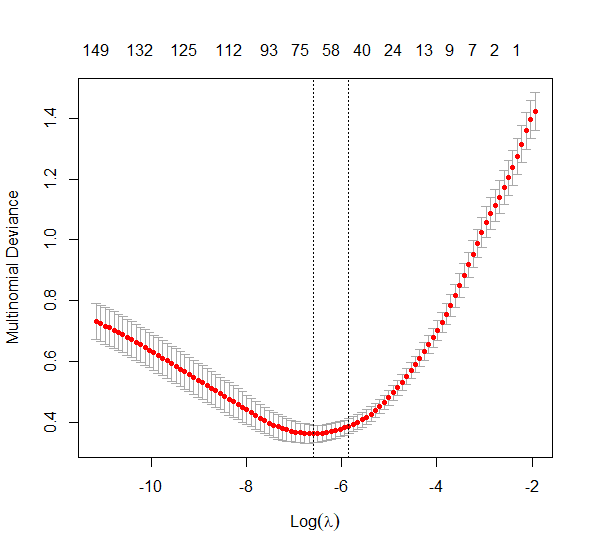

Supplement: S4 Fig — (TIFF) [file pone.0279739.s004.tiff]

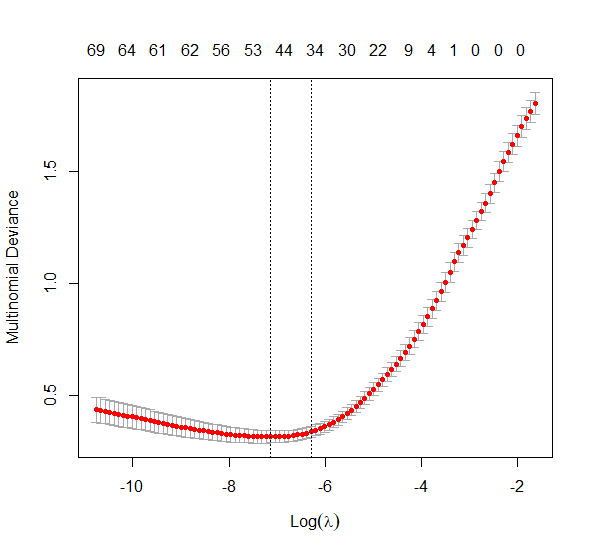

Supplement: S5 Fig — (TIFF) [file pone.0279739.s005.tiff]

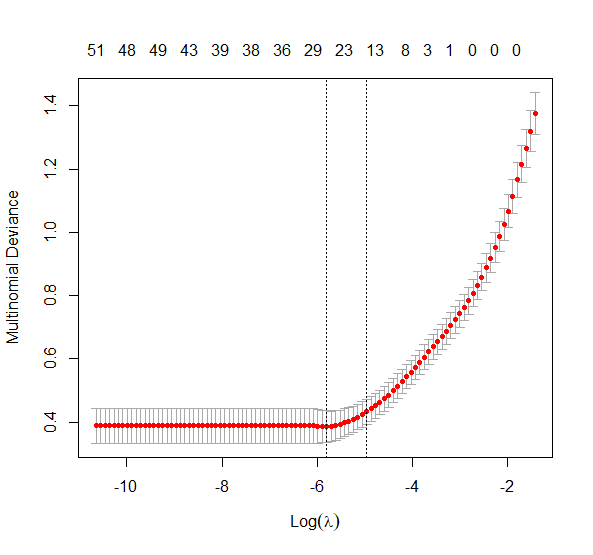

Supplement: S6 Fig — (TIFF) [file pone.0279739.s006.tiff]

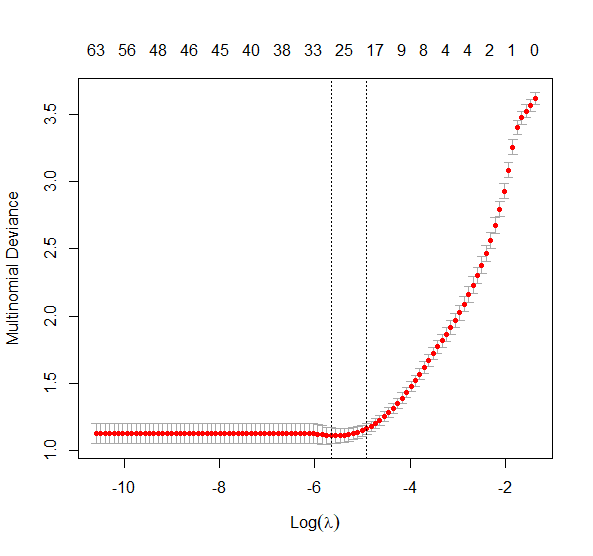

Supplement: S7 Fig — (TIFF) [file pone.0279739.s007.tiff]
