# Supplementary material for: Raman microspectroscopy and machine learning for use in identifying radiation-induced lung toxicity
Source: PLoS One. 2022 Dec 30;17(12):e0279739. doi: 10.1371/journal.pone.0279739 (PMC9803148; doi:10.1371/journal.pone.0279739)
Supplement: S1 Appendix — (PDF) [file pone.0279739.s008.pdf]

S8 Appendix. **Formula for multinomial deviance.** Let $y_{ig} = 1$ if observation i belongs to group g and $y_{ig} = 0$ otherwise. We fit a sparse logistic regression that estimates a vector of probabilities $\hat{p}(\mathbf{x}_i) = (\hat{p}_1(\mathbf{x}_i), ..., \hat{p}_G(\mathbf{x}_i))$ to each observation i, where $\hat{p}_g(\mathbf{x}_i)$ is the estimated probability of observation i belonging to group g. The deviance of this model is then expressed as

$$-2\sum_{i=1}^{n}\sum_{g=1}^{G} y_{ig}\log(\frac{\hat{p}_g(\mathbf{x}_i)}{y_{ig}}),$$

where n is the total number of training observations and G is the total number of groups.
